# Study of Charged Nanodroplet Deposition into Microcavity Through Many-Body Dissipative Particle Dynamics

**DOI:** 10.3390/mi16030278

**Published:** 2025-02-27

**Authors:** Yiwei Jin, Jiankui Chen, Wei Chen, Zhouping Yin

**Affiliations:** The State Key Laboratory of Intelligent Manufacturing Equipment and Technology, School of Mechanical Science and Engineering, HuaZhong University of Science and Technology, Wuhan 430074, China; jinyiwei@hust.edu.cn (Y.J.); yinzhp@hust.edu.cn (Z.Y.)

**Keywords:** E-jet printing, MDPD method, mesoscale, high-resolution display

## Abstract

For a near-eye display, a resolution of over 10,000 pixels per inch (PPI) for the display device is needed to eliminate the “screen door effect” and have better display quality. Electrohydrodynamic (EHD) printing techniques, which have the advantages of a high resolution, wide material applicability and flexibility in patterning, have been widely used in the printing of high-resolution structures. However, due to factors such as the extremely small size of the droplets, the electric charge, the electric field, and the unavoidable positioning error, various deposition defects can occur. For droplets at a nanoscale, the dynamic deposition process is hard to observe. The continuum hypothesis fails and the fluid cannot be described by the traditional Navier–Stokes equation. In this work, the behaviors of charged nanodroplet deposition into a microcavity in an electric field are studied. The many-body dissipative particle dynamics (MDPD) method is used to examine the deformation of the nanodroplet during the impact process at a mesoscale. The dynamic process of charged droplet deposition into a microcavity under an electric field is revealed. Strategies for failure-free printing are proposed by analyzing the influences of the impact speeds, positioning errors, charge levels and electric intensities on the out-of-pixel spread length. The relationship between the internal charge moves and the deformation of the charged droplet in the deposition process is first discussed. The spreading theory of charged droplet deposition into a microcavity with a positioning error is established by analyzing the Coulombic capillary number. Moreover, the printing parameter space that results in successful printing is acquired.

## 1. Introduction

**Figure 1:**
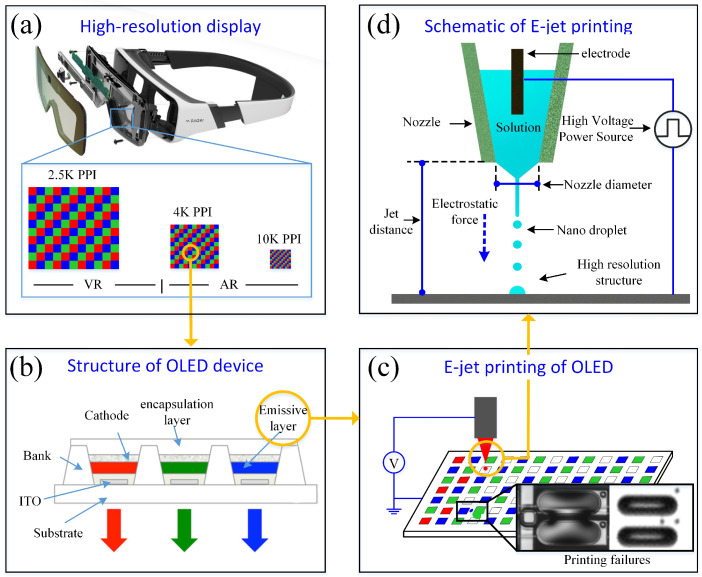
High-resolution display and EHD process: (**a**) high-resolution display, (**b**) structure of the OLED device, (**c**) E-jet printing of the OLED and printing failures, and (**d**) schematic of E-jet printing.

High-resolution organic light-emitting diode (OLED) displays [1] are widely used in AR/VR applications (as shown in Figure 1a,b). In order to achieve better visual effects, the requirements of the resolution for display devices are getting higher and higher. For a near-eye display, when the distance between the eyes and the display panel is shorter than 0.8 cm, a resolution of over 10,000 pixels per inch (PPI) for the display device is needed to eliminate the “screen door effect” and have better display quality. At present, OLED devices are mainly prepared by traditional lithography and evaporation [2,3], the material utilization rate is low and an environment characterized by a high temperature and vacuum is required. The electrohydrodynamic (EHD) printing technique (as shown in Figure 1d), which deposits micro/nanostructures through a high electric force, has been widely used in manufacturing high-resolution displays owing to their fascinating characteristics of a high resolution (<200 nm) and wide material applicability (ink viscosity 1–10,000 cps) [4]. However, due to factors such as the extremely small size of the droplets, the electric charge, the presence in the electric field, and the unavoidable positioning error, various deposition defects, such as “bridge” and “scatters”, can occur (as shown in Figure 1c) [5,6]. Therefore, it is critical to reveal the mechanisms of micro/nanodroplet deposition to improve the uniformity and accuracy of printed high-resolution display devices.

While investigations of droplet deposition behaviors have been conducted for more than 100 years, the phenomenon is still far from being fully understood [7]. A lot of research has been conducted on neutral micro/nanodroplet deposition. Gomaa et al. [8] experimentally analyzed the impact behavior of cloud-sized microdroplets and macrodroplets upon superhydrophobic surface (SHS) impact, and a preliminary theoretical model based on the energy balance and accounting for the substrate hysteresis was proposed. Pan et al. [9] established thermodynamic assumptions regarding the growth of condensation droplets and a mathematical formulation of the droplet energy functionals, and the research presented a detailed analysis of the factors affecting the surface condensation and heat transfer. Xu et al. [10] studied the process of a nanodroplet’s impact on a micro-structured surface. The effects of the micro-column height, surface wettability and gravity on the droplet wetting state were analyzed. Hasan et al. [11] investigated the impact of a nanodrop on a solid surface and the consequent deformation using the volume-of-fluid method, and the developed model had excellent agreement with the experimental results.

In the EHD printing process, the electric field and droplet charge play an important role in droplet deposition as they can significantly modify the morphology of the impacting droplets. Ghazian et al. [12] investigated the dynamics of the spread and impact of a dielectric droplet on a dry conductive substrate in the presence of an external vertical electric field and found that the droplet’s maximum spreading diameter and the rate of spread increase with an increasing surface charge density. Liu et al. [13] performed molecular dynamics studies to reveal the bouncing dynamics of a nanodroplet impacting a hydrophobic surface under electric fields with various field strengths and directions. Shen et al. [14] used a 3D diffuse interface model to simulate the impact of a dielectric droplet under the influence of a horizontal electric field, finding that the horizontal electric field can be useful to suppress splashing by reducing the rising angle made by the lamella with the substrate. Xu et al. [15] experimentally investigated the effects of an electric field on the droplet impact behavior in different thermal regimes and the subsequent dynamic mechanisms. A textured surface helps improve the uniformity of the deposited droplets due to the unavoidable positioning error. Liou et al. [16] performed micro-flow visualization and computational fluid dynamics studies complementarily to study microcavity deposition phenomena.

The physics embedded in the deposition of droplets into microcavities has also been addressed. Zhang et al. [17,18] investigated the dynamics of inkjet deposition into square microcavities utilizing a three-dimensional multi-relaxation-time model, and the effects of the wettability, density ratio, droplet viscosity and impact velocity were explored to reveal the droplet–microcavity interactions. Jackson et al. [19] investigated the dynamics of a single droplet deposited into a confined space and its final equilibrium morphology, with an emphasis on droplet deposition under print head misalignment, the effect of nonuniform wettability, and the deposition of droplets with varying sizes. Zhang et al. [20] numerically studied the deposition of nanoparticle-laden multi-droplets on a textured surface, and the simultaneous and successive impact modes were discussed.

When the diameter of a droplet decays to nanometers, the dynamic deposition process is hard to observe and the continuum hypothesis fails and the fluid cannot be described by the traditional Navier–Stokes equation. The impact mechanism of charged nanodroplet deposition into a microcavity is still unclear. In this work, the behaviors of charged nanodroplet deposition into a microcavity in an electric field are studied. The many-body dissipative particle dynamics (MDPD) method is used to examine the deformation of the nanodroplet in the impact process at a mesoscale. The deformation processes of the out-of-pixel spread at different charge levels, different electric intensities and different positioning errors are simulated. The relationship between the internal charge moves and the deformation of the charged droplet in the deposition process is first discussed. Moreover, the spreading theory and the printing parameter space that results in successful printing are established.

## 2. Methodology and Model

**Figure 2:**
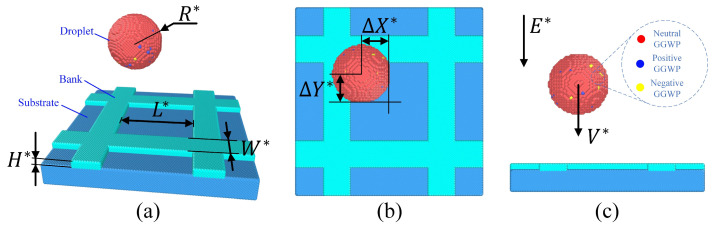
The scenario of the initial condition: (**a**) 3D view, (**b**) vertical view, and (**c**) side view.

The scenario where a liquid droplet is placed above the microcavity is illustrated in Figure 2a,b. The lattice model is set as face-centered cubic (fcc). $R^{*}$ is the radius of the nanodroplet, including the neutral coarse-grained particle (CGPs), positive CGPs, and negative CGPs, as shown in Figure 2c. The numbers of positive CGPs and negative CGPs are $N_{p}$ and $N_{n}$. $L^{*}$ is the side length of the square microcavity. $H^{*}$ and $W^{*}$ are the depth of the cavity and width of the bank. ${∆X}^{*}$ and ${∆Y}^{*}$ are the droplet positioning errors in the x direction and y direction, respectively. $V^{*}$ is the droplet impact speed. $E^{*}$ is the electric field intensity.

The MDPD method is suitable for simulating the droplet deposition dynamics of neutral particles at a mesoscale. Its interaction force ($F_{ij}$) is defined as the sum of the conservative force ($F_{ij}^{C}$), the dissipative force ($F_{ij}^{D}$), and the random force ($F_{ij}^{R}$) [21]. The conservative force is calculated as follows:

(1) $$F_{ij}^{C}=A^{*}\omega_{c}\left(r_{ij}^{*}\right)e_{ij}^{*}+B^{*}\left(\rho_{i}^{*}+\rho_{j}^{*}\right)\omega_{d}\left(r_{ij}^{*}\right)e_{ij}^{*}$$

(2) $$\omega_{c}\left(r_{ij}^{*}\right)=1-r_{ij}^{*}/r_{c}^{*}$$

(3) $$\omega_{d}\left(r_{ij}^{*}\right)=1-r_{ij}^{*}/r_{d}^{*}$$

where the first term with a negative coefficient $A^{*}$ < 0 stands for an attractive force, and the second term with $B^{*}$ > 0 is the density-dependent repulsive force, $e_{ij}^{*}=r_{ij}^{*}/r_{ij}^{*}$ is the unit vector, and $r_{c}^{*}$ and $r_{d}^{*}$ are the interaction ranges. $\omega_{c}\left(r_{ij}^{*}\right)$ and $\omega_{d}\left(r_{ij}^{*}\right)$ are weight functions. $\rho_{i}^{*}$ is the local number density of particle i, and $\rho_{j}^{*}$ is the local number density of particle j. The dissipative force and random forces are given by:

(4) $$F_{ij}^{D}=-\gamma^{*}\omega^{D}\left(r_{ij}^{*}\right)\left(e_{ij}^{*}\cdot v_{ij}^{*}\right)e_{ij}^{*}$$

(5) $$F_{ij}^{R}=-\sigma^{*}\omega^{R}\left(r_{ij}^{*}\right)\xi_{ij}∆t^{-1/2}e_{ij}^{*}$$

where $v_{ij}^{*}=v_{i}^{*}-v_{j}^{*}$ and $e_{ij}^{*}=r_{ij}^{*}/r_{ij}^{*}$. $\gamma^{*}$ and $\sigma^{*}$ are the intensity of the dissipative and random forces, respectively. $\omega^{D}\left(r_{ij}^{*}\right)$ and $\omega^{R}\left(r_{ij}^{*}\right)$ are the weight functions, and $\xi_{ij}$ are the symmetric Gaussian random variables. The fluctuation dissipation theorem is used to maintain the equilibrium temperature:

(6) $${\left(\sigma^{*}\right)}^{2}=2\gamma^{*}k_{b}^{*}T^{*}$$

(7) $$\omega^{D}\left(r_{ij}^{*}\right)={\omega^{R}\left(r_{ij}^{*}\right)}^{2}={\left(1-r_{ij}^{*}/r_{c}^{*}\right)}^{2}$$

where $k_{b}^{*}$ and $T^{*}$ are Boltzmann’s constant and the absolute temperature at a reduced unit, respectively.

As the droplet charge and external electric field are considered, the Coulombic force between two charged CGPs is calculated as follows:

(8) $$F_{ij}^{Coul}=\frac{Cq_{i}^{*}q_{j}^{*}}{{\left(r_{ij}^{*}\right)}^{2}}e_{ij}^{*}\;\mathrm{where}\;r_{ij}^{*}<r_{coul}^{*}$$

where C is the energy-conversion constant, $q_{i}^{*}$ and $q_{j}^{*}$ are the charges of the charged CGPs, and $r_{coul}^{*}$ is the cutoff radius of the Coulombic force. The electric field force $F_{i}^{E}$ of a charged CGP under electric field $E^{*}$ is written as follows:

(9) $$F_{i}^{E}=q_{i}^{*}E^{*}$$

Accordingly, the CGP’s motions can be calculated as follows:

(10) $${\dot{r}}_{i}^{*}=v_{i}^{*}$$

(11) $$m_{i}=F_{ij}=\sum_{i\neq j}\left(F_{ij}^{C}+F_{ij}^{D}+F_{ij}^{R}+F_{ij}^{Coul}\right)+F_{i}^{E}$$

where $r_{i}^{*}$ is the motion vector of particle i, and $m_{i}$ is the mass of particle i.

At the macroscale, a fluid is characterized by properties such as the density, viscosity, surface tension and electrical conductivity. However, at the mesoscale, a fluid particle is characterized by the attractive and repulsive coefficients $A^{*}$ and $B^{*}$, interaction ranges $r_{c}^{*}$ and $r_{d}^{*}$, dissipative coefficient $\gamma^{*}$, number density $\rho^{*}$, and charge level $q^{*}$. These mesoscale parameters are often expressed in reduced units and labeled by asterisks. It is important to acquire the physical units of the length ($l_{unit}$), mass ($m_{unit}$), and time ($t_{unit}$) in relation to a specific set of properties of a real liquid. According to the dimensional analysis [22]:

(12) $$m_{unit}={l_{unit}}^{3}\frac{\rho }{\rho^{*}}$$

(13) $$t_{unit}={\left(m_{unit}\frac{\Gamma^{*}}{\Gamma }\right)}^{0.5}$$

(14) $$\frac{{l_{unit}}^{2}}{t_{unit}}=\frac{\varphi }{\varphi^{*}}$$

where Γ, ρ, φ are the surface tension coefficient, density, and kinematic viscosity of the physical fluid. The same properties of the surface tension coefficient, density, and viscosity of the MDPD fluid are expressed in reduced units and labeled with an asterisk.

According to Equations (12)–(14), to acquire the parameters of the MDPD fluids, the physical units of the length ($l_{unit}$), mass ($m_{unit}$), and time ($t_{unit}$) should be obtained. $m_{unit}$ is the mass of a coarse-grained particle.

The physical unit of the length ($l_{unit}$) can be calculated as follows:

(15) $$l_{unit}=\sqrt[{3}]{\frac{\rho^{*}m_{unit}}{\rho }}$$

where $k_{B}T$ is usually chosen as the energy unit, and based on that, most researchers choose thermodynamic velocity as the speed unit of the system:

(16) $$V_{unit}=\sqrt{\frac{k_{B}T}{m_{unit}}}$$

where $k_{B}$ is the Boltzmann constant, and T is the temperature of the system. $k_{B}=1.380649^{-23\;}J/K$, T=298 K.

Then, the physical unit of the time can be acquired:

(17) $$t_{unit}=\frac{l_{unit}}{V_{unit}}$$

The surface tension coefficient, density, and viscosity of the MDPD fluid can be calculated from Equations (12)–(17). The kinetic viscosity [19] and surface tension [20] in reduced units are expressed as follows:

(18) $$\varphi^{*}=\frac{45k_{b}^{*}T^{*}}{4\pi \gamma^{*}\rho^{*}{r_{c}^{*}}^{3}}+\frac{2\pi \gamma^{*}\rho^{*}{r_{c}^{*}}^{5}}{1575}$$

(19) $$\Gamma^{*}=-\frac{\pi }{240}\left(0.42A^{*}{r_{c}^{*}}^{5}{\rho^{*}}^{2}+0.003B^{*}{r_{d}^{*}}^{5}{\rho^{*}}^{3}\right)$$

In this work, as a typical solvent in OLED applications, the deposition of a charged ethyl benzoate drop is studied. The density, viscosity and surface tension of ethyl benzoate are 1045 $kg/m^{3}$, $2.1\times 10^{-6}\;m^{2}/s$ and 0.035 N/m, respectively. The corresponding MDPD parameters can be calculated by Equations (10)–(17), which are listed in Table 1. All the parameters are expressed in reduced units in this work, while the conversion of the MDPD parameters to physical parameters is not discussed hereafter.

**Figure 3:**
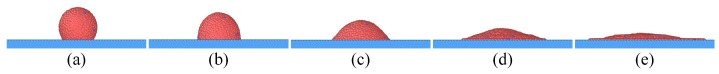
The equilibrium outcomes of a sessile droplet on substrates with different contact angles θ under different $A^{*}$. (**a**) θ=128°, $A^{*}=-25$, (**b**) θ=90°, $A^{*}=-45$, (**c**) θ=70°, $A^{*}=-65$, (**d**) θ=28°, $A^{*}=-70$, and (**e**) θ=11°, $A^{*}=-85$.

A textured surface with a hydrophobic bank and hydrophilic substrate is usually used in industry to improve the uniformity of deposited droplets. In the MDPD system, the wettability of the substrate is tunable by appropriately setting the intensity of attractive force $A^{*}$. The equilibrium outcomes of a sessile droplet on substrates with different contact angles are acquired under different $A^{*}$, as shown in Figure 3a–e. The MDPD parameters between the droplet and the bank, and between the droplet and the substrate, are listed in Table 2.

The open-source molecular dynamics package LAMMPS is used to run the simulation, and the post-processing of the results is performed using the visualization tool OVITO-3.10.6. The pair style of MDPD is adopted and the time step is set to 0.001 $t_{unit}$ in the simulation. The time to reach an equilibration state is set as 10 $t_{unit}$. The default calculation parameters for the simulation are listed in Table 3. In the printing process, the droplet positioning error is mainly caused by the jet timing in the printing direction (x direction); for the convenience of analysis, the positioning error in the y direction is set to zero. The droplets ejected under a positive electric field mainly have a positive charge and the number of negative CGPs in the MDPD system is ignored. Hereafter, the parameters listed in Table 3 are used in all cases unless otherwise specified.

## 3. Results and Discussion

In the printing of high-resolution (>10,000 PPI) OLEDs using the EHD technique, the deposition accuracy is hard to control at the micro/nanoscale due to factors such as the extremely small size of the droplets, the electric charge and the electric field. In this section, the deposition of a charged nanodroplet with a certain positioning error is investigated. The deformation processes of the out-of-pixel spread at different positioning errors, impact speeds, charge levels and electric intensities are simulated. The printing theory and the printing parameter space that results in success printing are established.

### 3.1. Description of the Morphologies of the Charged Nanodroplet Deposition

**Figure 4:**
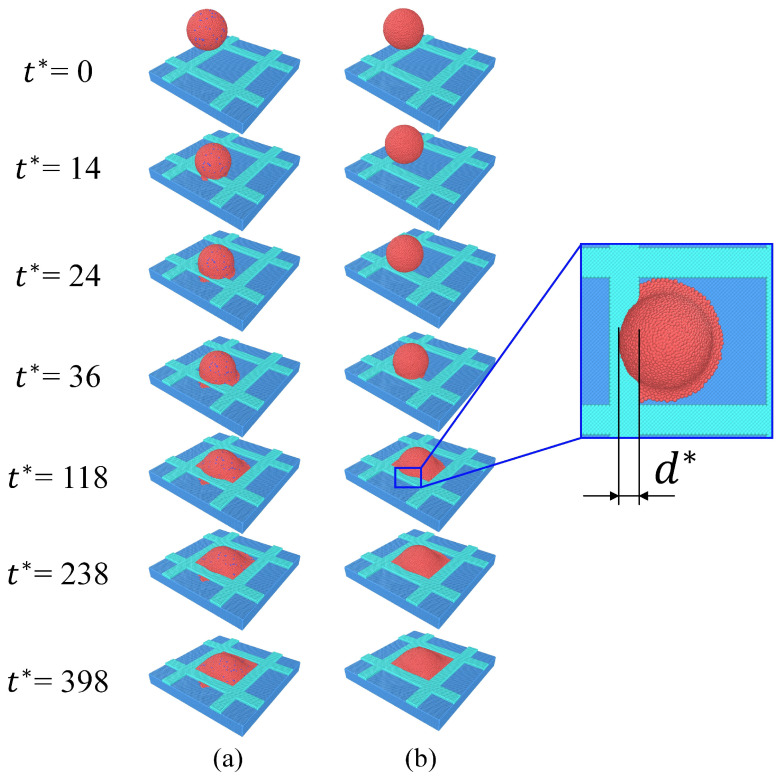
Simulation results of nanodroplet deposition into a microcavity with a positioning error: (**a**) charged droplet under an electric field, and (**b**) neutral droplet without an electric field.

In this section, the behavior of charged nanodroplet deposition into a dry microcavity is numerically studied. To intuitively understand the differences in the deposition process between charged nanodroplets and neutral nanodroplets at the mesoscale, the morphology and dynamics are analyzed in this section. The simulation results of nanodroplet deposition into a microcavity with the positioning error ${∆x}^{*}$ = 25 are shown in Figure 4. In Figure 4a, the charged nanodroplet is placed in a uniform electric field with $q^{*}=1$ and $E^{*}=10$. In Figure 4b, the droplet is neutral with $q^{*}=0$ and $E^{*}=0$. In the case of the neutral droplet, at the initial stage, due to the relatively large positioning error, the nanodroplet impinges on the bank and starts to spread under the influence of the inertial forces. Due to the differences between the bank and the pixel in terms of the contact angle and height, the droplet spreads faster in the pixel pit. Since the occurrence of printing failures is mostly related to the spreading behavior outside the pixel, we will not discuss the spreading of droplets inside the pixel pit too much. After the out-of-pixel spreading length $d^{*}$ reaches its maximum, it begins to retract and is eventually dragged back into the pixel. In the case of the charged droplet, due to the existence of an electric field and charged particles, the charged droplet impinges on the bank earlier and more severe spread occurs compared with the neutral droplet. It spreads over the whole bank and is attached to the bottom of the neighboring pixel; thus, a “temporary bridge” is formed. As the spread continues, the connection between the two pixels breaks and much of the droplet is dragged back to the right pixel, while a bit of the droplet is left in the neighboring pixel. In the printing of the OLED, different materials are needed in the neighboring pixels. When the amount of out-of-pixel spread is larger than the bank width, the materials in the pixel will be polluted or the volume of liquid will be changed, which will cause display defects and needs to be avoided in the printing of high-resolution OLEDs.

### 3.2. Influence of Different Parameters on Out-of-Pixel Spreading Length

#### 3.2.1. Influence of Droplet Positioning Error

**Figure 5:**
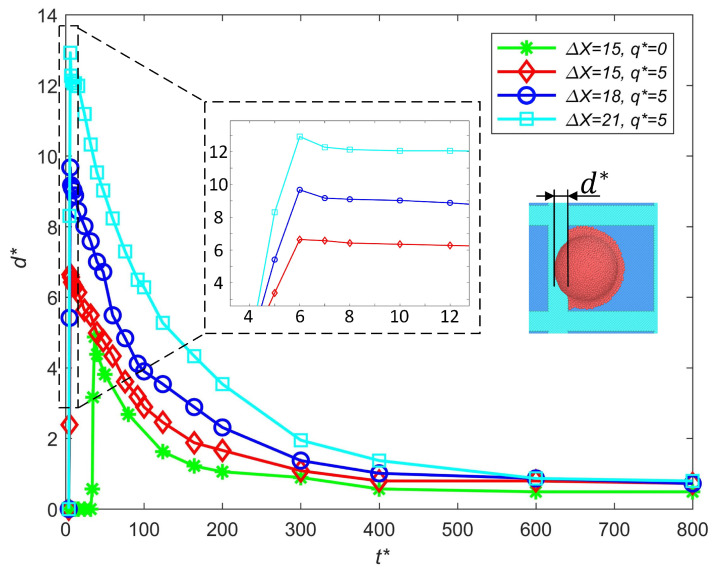
Influence of the droplet positioning error on the out-of-pixel spreading length.

The influence of the droplet positioning error on the out-of-pixel spreading length is shown in Figure 5. Compared with the neutral droplet, the existence of a charge will enlarge the out-of-pixel spread with the same positioning error. The out-of-pixel spreading length increases with the positioning error, while it takes almost the same amount of time for the out-of-pixel spreading length to reach the maximum. When the droplet positioning error increases from 15 to 18, the increment of the out-of-pixel spreading $d^{*}$ is 3.04. When the droplet positioning error increases from 18 to 21, the increment of the out-of-pixel spreading $d^{*}$ is 3.25. The out-of-pixel spreading length does not increase linearly with the droplet positioning error, as the increment of the out-of-pixel spreading length is also extended with the positioning error. The out-of-pixel spreading length is sensitive to the droplet positioning error. In order to avoid printing defects, it is necessary to strictly control the positioning error. The droplet positioning error is inevitable in the actual process of E-jet printing, although the maximum allowable positioning error can be acquired by analyzing the maximum out-of-pixel spreading length, which helps eliminate printing defects.

#### 3.2.2. Influence of Impact Speed

**Figure 6:**
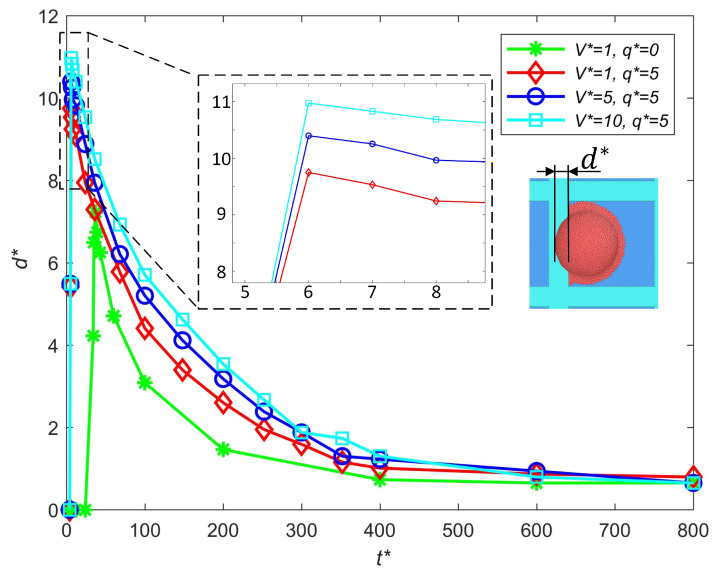
Influence of the impact speed on the out-of-pixel spreading length.

The influence of the droplet impact speed on the out-of-pixel spreading length is discussed in this section, as shown in Figure 6. Compared with the neutral droplet, the existence of a charge will enlarge the out-of-pixel spread at the same impact speed. As the droplets have the same initial position, the droplet with a higher impact speed impinges on the substate earlier. The out-of-pixel spreading length increases with the impact speed. The droplet with a higher impact speed has larger initial kinetic energy, and in the spreading process, the kinetic energy is converted to surface tension. When the out-of-pixel spreading length reaches its maximum, all the kinetic energy is converted to surface tension (a small portion of energy is dissipated by viscosity). Therefore, a higher impact speed will result in a larger out-of-pixel spread. To avoid excessive out-of-pixel spreading, an appropriate waveform is needed to make the impact of the droplet occur at a relatively low speed while maintaining stable ejection.

#### 3.2.3. Influence of Charge Levels

**Figure 7:**
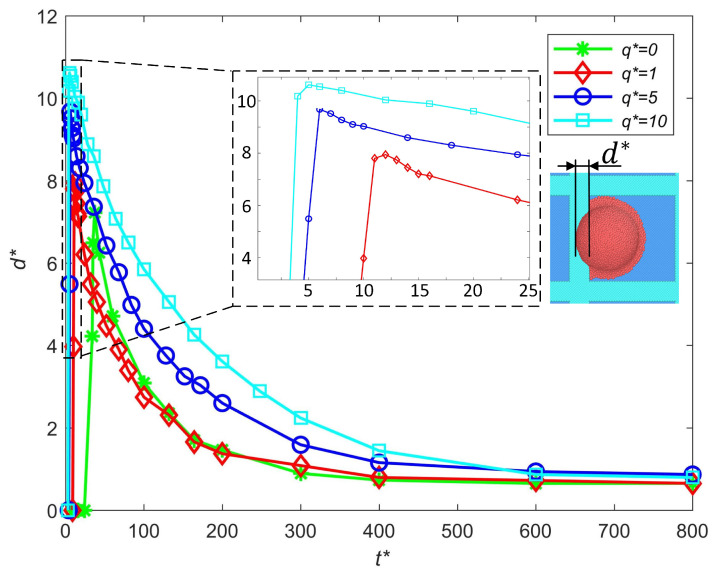
Influence of the charge level on the out-of-pixel spreading length.

In this section, the influence of the charge level on the out-of-pixel spreading length is analyzed. Droplets ejected under positive voltage usually have a positive net charge. For the MDPD model, the total charge of a droplet is $Q^{*}=N_{p}\times q^{*}$, where $q^{*}$ is the charge level of a single CGP. Here, we fix the number of positive CGPs and change the total charge of the droplet by changing the charge level of a single CGP $q^{*}$. The influence of the charge level on the out-of-pixel spreading length is shown in Figure 7. The charged droplets are set at the same initial position, so the droplet with a higher charge level will impact on the substrate earlier as it is subjected to greater electrical force. With the increase in the charge level, both the spreading speed and the out-of-pixel spreading length increase. The main reason that causes this phenomenon is that the existence of the positively charged CGPs decreases the surface tension of the droplet. Decreasing the surface tension will decrease the contact angel between the bank and the charged droplet; thus, the spreading process is enhanced. To avoid printing failure, a smaller intensity of electric field is preferred. In the E-jet printing process, the total charge of the ejected droplet is relative to the dielectric constant of the ink and the intensity of the electric field. Based on the premise of stable ejection, a larger dielectric constant of the ink and a smaller intensity of the electric field are better for failure-free printing.

#### 3.2.4. Influence of Electric Intensities

**Figure 8:**
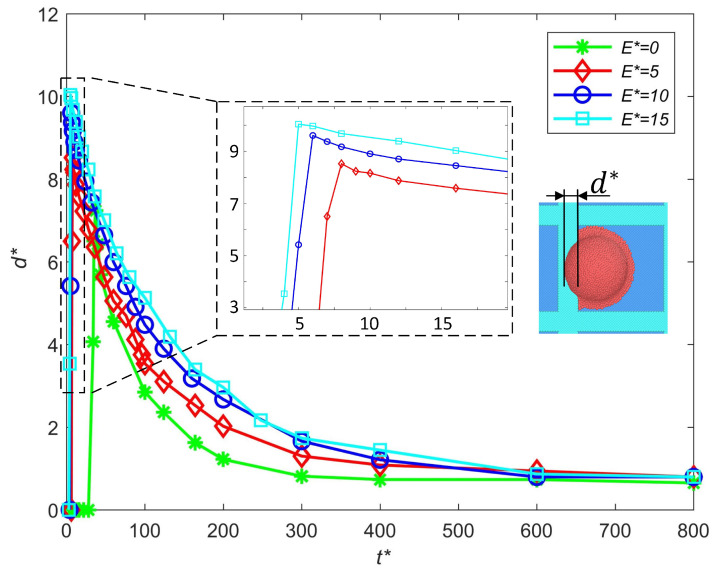
Influence of the electric field intensity on the out-of-pixel spreading length.

The influence of the electric field intensity on the out-of-pixel spreading length is discussed in this section, as shown in Figure 8. The influence of the electric field intensity is similar to that of the charge level. Due to the acceleration process of the charged droplet in the electric field, the droplet in a stronger electric field will contact the substrate earlier and gain greater kinetic energy at the moment of impact, thus causing more severe spreading. Meanwhile, larger electric field intensity will magnify the electrowetting effect, which also enhances the out-of-pixel spread. A smaller electric field intensity is beneficial for suppressing out-of-pixel spreading.

### 3.3. The Charge Moves and the Failure-Free Printing Parameter Space

**Figure 9:**
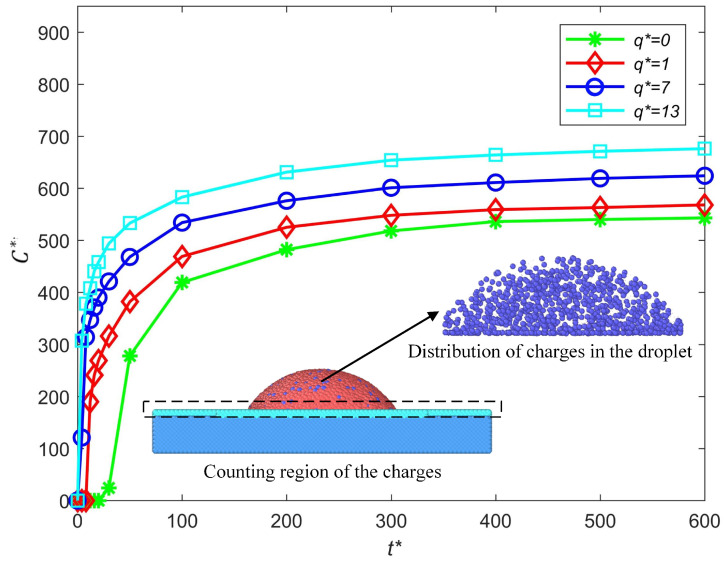
Influence of the charge level on the charge moves in the deposition process.

In the EHD printing of high-resolution displays, the droplet positioning error is usually inevitable due to the variation in the printing parameters and the motion of the axis; thus, increasing the maximum allowable droplet positioning error helps to achieve failure-free printing. Compared with the neutral droplet’s deposition, the main difference in terms of the charged nanodrop’s deposition is the existence of an electric charge and an electric field. The electric charge $q^{*}$ is applied to analyze the charge moves in the deposition process. As shown in Figure 9, the number of charges near the substrate $C^{*}$ is counted to indicate the movement of the charges under different electric charge $q^{*}$. The number of charges near the substrate increases in the impact and spread process, and it reaches its maximum when the droplet comes to equilibrium. We can find that more charges are driven toward the substrate with the increase in the electric charge $q^{*}$.

To obtain more general results, the electric Reynolds number ${Re}_{E}$ and the electric capillary number ${Ca}_{E}$ dimensionless parameters are often used to discuss the dynamics of charged liquids. The Coulombic capillary number is defined to analyze the comprehensive impact of the surface tension and the Coulombic force on the nanodroplet deposition process. The Coulombic capillary number given by:

(20) $${Ca}_{c}=\frac{{Re}_{E}}{{Ca}_{E}}=\frac{\epsilon^{*}\Gamma^{*}}{S^{*}\eta^{*}R_{0}}$$

(21) $$S^{*}=\frac{45c^{*}{q^{*}}^{2}}{2\pi \gamma^{*}\rho^{*}{r_{c}^{*}}^{3}}$$

where $\epsilon^{*}$ is the dielectric constant of ethyl benzoate. $\Gamma^{*}$ is the surface tension of the droplet. $\eta^{*}$ is the dynamic viscosity of the droplet. $c^{*}$ is the concentration, which is expressed by the percentage of charged particles. $q^{*}$ is the electric charge of a single particle. $\gamma^{*}$ is the intensity of the dissipative force. The dimensionless positioning error parameter is defined as $\alpha^{*}=\left({∆X}^{*}+R^{*}-L^{*}/2\right)/2R^{*}$, where 0 means that it is completely deposited inside the pixel, and 1 means that it is completely deposited outside the pixel. The dimensionless maximum out-of-pixel spreading length is calculated as $D^{*}=d_{max}^{*}/W^{*}$, and $D^{*}>1$ indicates that the maximum spread length exceeds the bank of the pixel during the deposition process, resulting in color mixing or “bridge” defects. The parameters for the calculation of ${Ca}_{c}$ are listed in Table 4.

**Figure 10:**
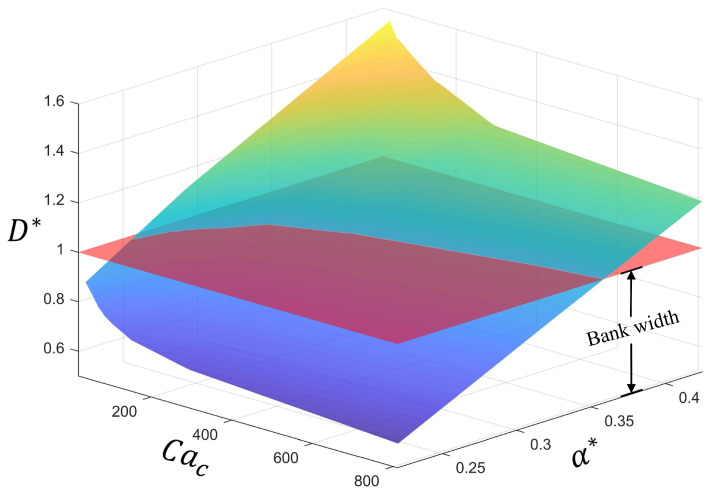
Influence of the Coulombic capillary number and positioning error on the maximum out-of-pixel spreading length.

The influence of the Coulombic capillary number and positioning error on the maximum out-of-pixel spreading length is shown in Figure 10. The maximum out-of-pixel spreading length increases with the increase in the positioning error. By contrast, it decreases with the increase in the Coulombic capillary number, which means that increasing the Coulombic force or decreasing the surface tension can enhance the out-of-pixel spread of a charged droplet.

With further analysis, the relationship between the Coulombic capillary number, positioning error and maximum out-of-pixel spreading length can be well fitted with an R-square of 0.9954.

(22) $$D^{*}=-0.1492+2.688{{Ca}_{c}}^{-\frac{1}{2}}+2.988\alpha^{*}$$

**Figure 11:**
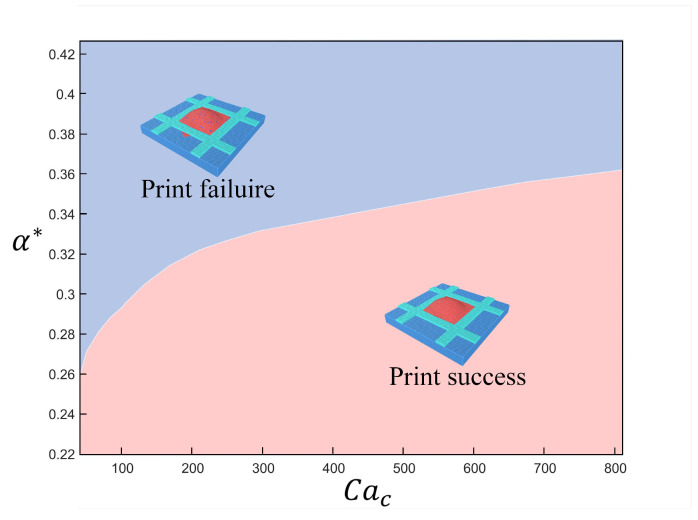
The failure-free parameter space.

By projecting the data in Figure 10 onto the XY plane, the failure-free printing parameter space can be acquired, which is shown in Figure 11. The maximum allowable positioning error increases with the increase in the Coulombic capillary number. To enlarge the allowable droplet positioning error, a relatively smaller electric force is preferred.

## 4. Conclusions

In this work, the many-body dissipative particle dynamics (MDPD) method has been used to study the behaviors of a charged nanodroplet with a positioning error during deposition into a microcavity in an electric field at the mesoscale. The MDPD parameters in relation to a specific set of properties of ethyl benzoate are acquired by dimensional analysis. The deformation processes of the out-of-pixel spread at different positioning errors, impact speeds, charge levels and electric intensities at the mesoscale have been simulated. The main conclusions are as follows:

- Strategies for failure-free printing are proposed, where increasing the impact speed will enlarge the out-of-pixel spreading length due to an increase in the initial kinetic energy. Increasing the charge level and electric field intensity will both increase the out-of-pixel spreading length by decreasing the surface tension and enhancing the electrowetting effect. To avoid excessive out-of-pixel spreading, an appropriate waveform is needed to make the impact of the droplet occur at a relatively low speed while maintaining stable fracture. Based on the premise of stable ejection, a larger dielectric constant of the ink and a smaller intensity of the electric field are better for failure-free printing.
- The relationship between the internal charge moves and the deformation of the charged droplet in the deposition process is first discussed. The electric force changes the distribution of the charges in the droplet and more charges are driven toward the substrate with the increase in the electric force of the charged particles.
- The spreading theory of charged droplet deposition into a microcavity with a positioning error is established by analyzing the Coulombic capillary number. Moreover, the printing parameter space that results in successful printing is acquired.

For the printing of a large area of high-resolution displays, the vibration of the substrate will reduce the printing accuracy, and as the droplet size is extremely small, the disturbance of the airflow in the printing area cannot be ignored. The influence of charge accumulation in the substrate and the crosstalk effect makes the problem of droplet deposition more complicated, which needs further investigation.

## Figures and Tables

**Table 1:** MDPD parameters for ethyl benzoate.

$A^{*}$	$B^{*}$	$r_{c}^{*}$	$r_{d}^{*}$	$r_{coul}^{*}$	$\rho^{*}$	$\gamma^{*}$	$k_{b}^{*}T^{*}$	$l_{unit}$ (nm)	$t_{unit}$ (ns)	$V_{unit}$ (nm/ns)
−209.5	1870	3	1.5	8	0.8	99	1	4.58	0.802	5.715

**Table 2:** MDPD parameters between the droplet and the bank, and between the droplet and the substrate.

Parameters	$A^{*}$	$B^{*}$	$r_{c}^{*}$	$r_{d}^{*}$	$r_{coul}^{*}$	$\rho^{*}$	$\gamma^{*}$	$k_{b}^{*}T^{*}$
Between droplet and bank	−45	1200	3.6	1.8	8	0.8	99	1
Between droplet and substrate	−85	1200	3.6	1.8	8	0.8	99	1

**Table 3:** The default calculation parameters.

$R^{*}$	$L^{*}$	$H^{*}$	$W^{*}$	${∆X}^{*}$	${∆Y}^{*}$	$V^{*}$	$E^{*}$	$q^{*}$	$N_{p}$	$N_{n}$
14.25	44	2	9	18	0	1	10	5	976	0

**Table 4:** Parameters for the calculation of ${Ca}_{c}$.

Parameters	$\epsilon^{*}$	$\Gamma^{*}$	$\eta^{*}$	$R^{*}$	$c^{*}$	$q^{*}$	$\gamma^{*}$	$\rho^{*}$	$r_{c}^{*}$
Value	6	178	61.36	14.25	0.05	3~19	99	0.8	3

## Data Availability

The original contributions presented in this study are included in the article.

## References

[1] Joo W.J., Kyoung J., Esfandyarpour M., Lee S.-H., Koo H., Song S., Kwon Y.-N., Song S.H., Bae J.C., Jo A. (2020). Metasurface-driven OLED displays beyond 10,000 pixels per inch. Science.

[2] Kim S.K. (2016). Effect of Computational Lithography Parameters on the Organic Light-Emitting Diodes. J. Nanosci. Nanotechnol..

[3] Jun S., Kim M., Kim S.H., Lee M.Y., Lee E.K. (2013). A study on the evaporation process with multiple point-sources. Electron. Mater. Lett..

[4] Yin Z.P., Wang D., Guo Y., Zhao Z., Li L., Chen W., Duan Y. (2023). Electrohydrodynamic printing for high resolution patterning of flexible electronics toward industrial applications. InfoMat.

[5] Yakunin S., Chaaban J., Benin B.M., Cherniukh I., Bernasconi C., Landuyt A., Shynkarenko Y., Bolat S., Hofer C., Romanyuk Y.E. (2021). Radiative lifetime-encoded unicolour security tags using perovskite nanocrystals. Nat. Commun..

[6] Yang X., Yan Z.J., Zhong C.M., Jia H., Chen G., Fan X., Wang S., Wu T., Lin Y., Chen Z. (2023). Electrohydrodynamically printed high-resolution arrays based on stabilized CsPbBr3 quantum dot inks. Adv. Opt. Mater..

[7] Yarin A.L. (2006). Drop impact dynamics: Splashing, spreading, receding, bouncing…. Annu. Rev. Fluid Mech..

[8] Gomaa H., Tembely M., Esmail N., Dolatabadi A. (2020). Bouncing of cloud-sized microdroplets on superhydrophobic surfaces. Phys. Fluids.

[9] Pan X., Wang Y., Shen M. (2022). A conservative level set approach to non-spherical drop impact in three dimensions. Micromachines.

[10] Xu B., Zhang C., Chen Z., Yang Y., Cao Q. (2021). Investigation of nano-droplet wetting states on array micro-structured surfaces with different gravity. Comput. Fluids.

[11] Hasan M.N., Chandy A., Choi J.W. (2015). Numerical analysis of post-impact droplet deformation for direct-print. Eng. Appl. Comput. Fluid Mech..

[12] Ghazian O., Adamiak K., Castle G.S.P. (2014). Spreading and retraction control of charged dielectric droplets. Colloids Surf. A.

[13] Liu R., Wang Y.B., Yang S.W., Liu H.-W., Yang Y.-R., Wang X.-D., Lee D.-J. (2021). Impacting-bouncing nanodroplets on superhydrophobic surfaces under electric fields. Colloids Surf. A.

[14] Shen M., Li B.Q., Yang Q. (2023). A 3-D phase field study of dielectric droplet impact under a horizontal electric field. Int. J. Multiphase Flow.

[15] Xu H., Wang J., Yu K., Li B., Zhang W., Zuo L., Kim H.-B. (2022). Droplet impact on hot substrates under a uniform electric field. Phys. Fluids.

[16] Liou T.M., Chan C.Y., Shih K.C. (2009). Study of the characteristics of polymer droplet deposition in fabricated rectangular microcavities. J. Micromech. Microeng..

[17] Zhang L., Cheng X., Ku T., Song Y., Zhang D. (2018). Lattice Boltzmann study of successive droplets impingement on the non-ideal recessed microchannel for high-resolution features. Int. J. Heat Mass Transfer.

[18] Zhang L., Ku T., Cheng X., Song Y., Zhang D. (2018). Inkjet droplet deposition dynamics into square microcavities for OLEDs manufacturing. Microfluid. Nanofluid..

[19] Jackson F.F., Kubiak K.J., Wilson M.C.T., Molinari M., Stetsyuk V. (2019). Droplet misalignment limit for inkjet printing into cavities on textured surfaces. Langmuir.

[20] Zhang L., Wang X. (2021). Evaporation-driven nanoparticles motion and deposition on a textured surface in the inkjet process. Eng. Appl. Comput. Fluid Mech..

[21] Groot R.D., Warren P.B. (1997). Dissipative particle dynamics: Bridging the gap between atomistic and mesoscopic simulation. J. Chem. Phys..

[22] Arienti M., Pan W., Li X., Karniadakis G. (2011). Many-body dissipative particle dynamics simulation of liquid/vapor and liquid/solid interactions. J. Chem. Phys..

